# Evaluating a simple modified button approach in acromio-clavicular joint injury: Case report

**DOI:** 10.1016/j.ijscr.2025.110946

**Published:** 2025-01-27

**Authors:** Romy Deviandri, Dimas Harendra, Muhammad Hafiz Putratama, Ukhty Muslimah Syahrianti, Rima Farahdina, Muhammad Wiranata

**Affiliations:** a Department of Orthopedics, University of Groningen, University Medical Center Groningen, Groningen, the Netherlands; b Department of Surgery, Faculty of Medicine, Universitas Riau, Arifin Achmad Hospital, Pekanbaru, Indonesia; c Department of Orthopedics, Faculty of Medicine, Universitas Padjajaran, Hasan Sadikin Hospital, Bandung, Indonesia

**Keywords:** Shoulder joint, Surgery, Operative procedure, Questionnaire, Case report

## Abstract

**Introduction and importance:**

Shoulder stability is crucial, and appropriate therapy is essential for improving patient outcomes following an injury.

**Case presentation:**

A 30-year-old male was admitted to the clinic after lifting heavy objects at the fitness center. The physical examination reveals a decreased range of motion (ROM) and impaired flexion and external rotation of the shoulder joint. The left shoulder X-ray shows moderate soft tissue swelling without discontinuities of the bone, although elevation of the distal clavicle is prominent. After a comprehensive examination, the patient was diagnosed with an acromioclavicular (AC) joint injury.

**Clinical discussion:**

We use a mini-open surgical method called a Simple Modified Button Technique (SMB) to maintain shoulder function. The Visual Analog Scale (VAS) and Oxford Shoulder Score (OSS) were measured to evaluate the patient's outcome.

**Conclusion:**

The SMB procedure could adequately treat AC joint injuries. Measurement tools such as VAS and OSS have shown promising results. Adequate treatment will appropriately manage prolonged pain in AC joint injuries.

## Highlights

• A young male patient sustained an acute acromioclavicular (AC) joint injury.
• The surgical intervention performed was a simple modified button technique (SMB).
• The SMB is a simple and effective procedure with fewer complications for treating the AC injury case.
• The Oxford Shoulder Score and Visual Analog Scale (VAS) were used to measure outcomes.

## 1 Introduction

AC joint is a plane synovial joint that appears as the articulation between the scapula's acromion and the clavicle's acromial end. AC joint injuries are reported as common shoulder injuries and are more than 40 % of shoulder injuries, ranging from a simple strain to disruption of the ligaments [1]. AC joint injuries are five times more common in men aged 20–30 years, starting with pain symptoms, swelling in the shoulder, and rarely seen deformity [2]. Almost 10 % of these injuries are found in traffic accidents. Other literature also states explicitly that this injury often occurs in acceleration-deceleration events of traffic accidents [3].

Many experts treat AC joint injury cases based on the Rockwood and Green classification system, depending on the severity of the injury. In mild injuries (Type I- II), conservative management such as rest and physiotherapy is often sufficient for recovery. However, in more severe injuries (Type III-VI) involving significant ligament tears, surgical intervention may be required to restore joint stability and shoulder function. Recovery time varies; mild injuries usually require a few weeks, while more severe injuries may take several months, depending on the type of treatment received and the extent of tissue damage [4]. Minimal invasive procedure is a widely used technique using button instruments that excels through fixation strength and accelerates the range of motion (ROM) function. This technique needs a particular implant not provided in some healthcare centers. This case described a Simple Modified Button Technique (SMB) using a mini-plate with three holes and a non-absorbable suture, reported by using SCARE Guideline 2023 [5].

## 2 Case presentation

A 30-year-old male was admitted to the clinic after lifting heavy objects at the fitness center. He complained of aggravating pain in his left shoulder. One month before hospital admission, there was a history of pain in his left shoulder after being hit by a car while walking down the street and falling with his left shoulder hitting the ground. He experienced pain and difficulty moving his left shoulder. At that time, he consumed non-steroid anti-inflammatory drugs (NSAIDs) to reduce the pain and swelling. The complaint was declined without further treatment yet. The patient has been a smoker for the past five years and has no history of allergies or long-term medication use. His family has no history of sickness, and he works now as a courier. All his vital signs are typical. However, the patient experienced aggravating pain with a VAS score of 8. The localized pain and swelling affect his ability to move his left shoulder. The physical examination reveals decreased ROM, impaired flexion, and external shoulder joint rotation. The relevant Apley scratch test shows a restriction.

**Fig. 1:**
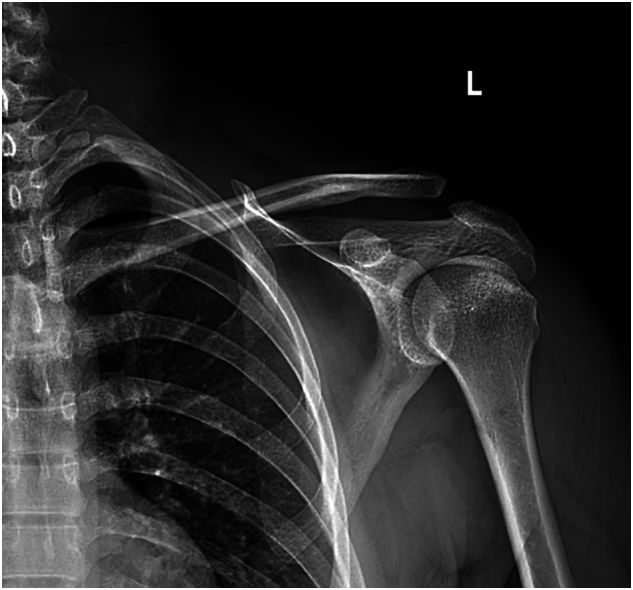
Shoulder X-ray shows a superior displacement of the clavicle above the superior border of the acromion.

The shoulder X-ray shows a superior displacement of the clavicle above the superior border of the acromion. After a thorough examination, the patient was diagnosed with an AC joint injury Rockwood classification type III (Fig. 1).

**Fig. 2:**
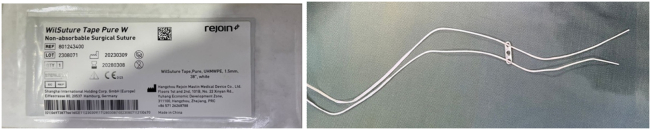
A non-absorbable multifilament suturing kit.

Our goals for this patient are to minimize the pain and improve his left shoulder's function. After agreeing with the informed consent, the patient underwent surgery with the SMB procedure to repair the AC joint. So far, there is no underlying disease that affected our surgery. An expert orthopedic surgeon can implement this procedure in our general hospital. We also prepare surgical instruments, including the AC joint aiming device, a non-absorbable multifilament sutures (Rejoin™), and one miniplate with three holes as a button fixation (Fig. 2).

**Fig. 3:**
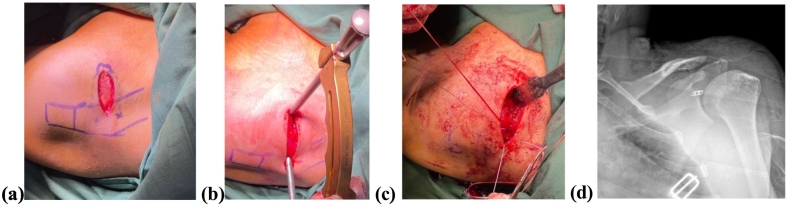
A marking of AC joint area (a). The aiming device was set up (b) and tightened using the Simple Modified Button Technique (c). The anatomical position was shown after surgery (d).

The patient was given the antibiotic–Ceftriaxone two grams intravenously to prevent infection during surgery. The patient is supine, with a sandbag in the affected shoulder. After an aseptic and antiseptic procedure, a Saber incision was performed adequately. The aiming device was set up at the coracoid's base and the distal clavicle– about 2.5–3 cm from the AC joint. A drill bit of 2.8 mm was used to drill the hole, and then the SMB procedure was figured out. The “button” was inserted through the hole and was fixed at the coracoid's base, and the suture was knotted at the clavicle with adequate strength, guided by the C-arm to maintain the anatomical position after fixation (Fig. 3).

After surgery, the patient's hemodynamic status remains stable, so intensive care management is unnecessary. The surgeon also advised the patient to control the healing process of the wound every week. He was also referred to a rehabilitation consultant to optimize the function of his left shoulder.

We assessed the patient's outcomes, particularly in his left shoulder, using the patient-reported outcome measurement tools: the Visual Analog Scale (VAS) to evaluate pain level and the Oxford Shoulder Score to evaluate functional improvement. These measurements were taken before, after, and six months post-surgery to track the healing process. The VAS score was 8 before the surgery, and the Oxford Shoulder Score was 24.

The success of the therapy depends on modifying the patient's lifestyle and ensuring optimal function. The physiotherapist instructed the patient to implement the shoulder pendulum exercise in the first month and avoid shoulder active range of motion. A month after surgery, the patient still felt mild pain, with a VAS score of three and an OSS of 30. In the second month, a small active range of motion exercise should be initiated, and a small weight property of less than a half kilogram could be allowed. The progression strengthening exercise was continued after three months, with the target of full ROM in all directions: flexion, extension, and rotation. After six months, the complaints declined, the level of VAS fell to 0, and the Oxford Shoulder Score was 46. The patient could perform a full active range of motion in all directions of the shoulder without limitation. So far, there have been no complications due to our procedure, and the patient's condition is improving with an additional physical examination.

The patient has given informed consent for publication, including photographic documentation.

## 3 Discussion

This case report aims to evaluate the outcomes of a patient with AC joint injuries treated using the SMB procedure. Acceptable outcomes after fixation and rehabilitation six months after surgery were found. Based on the VAS and OSS scores, a substantial result is achieved.

Most AC joint injury patients experienced significant shoulder pain and swelling. Pain mainly occurred during attempts to elevate the upper arm. In addition, tenderness is observed upon palpation of the shoulder [2]. Most patients also had limited ROM due to pain attempting to perform movement [2,6]. Based on that, using VAS and OSS scores to evaluate the outcomes is acceptable.

The therapy for patients with AC joint injuries is based on the severity level using the Rockwood and Green classification. Types I and II represent conditions where the AC ligament experiences a tear, but the coracoclavicular (CC) ligament remains intact. In these types, the injury is caused by low-impact forces. Hence, conservative management involving sling immobilization, analgesics, ice, and physical therapy with active and passive movements is often accepted [1,2]. This management aims to reduce stress on the injured ligaments and accelerate the healing process. Engaging in heavy activities is not permitted until the pain decreases and the ROM of the joint improves [6].

In AC joint injuries classified as types III, IV, V, and VI, the AC and CC ligaments experience tears, with the distal clavicle exhibiting posterior and inferior displacement. These types of injuries result from higher impact forces. Surgical interventions that can be performed include CC screw fixation, hook plate fixation, button CC fixation, and ligament reconstruction using the Weaver-Dunn procedure [6]. There is no gold standard in the treatment of AC joint injuries. CC screw fixation involves performing an open reduction on the dislocated AC joint, and the screws are placed between the coracoid process and the distal clavicle. This technique has drawbacks, including the need for screw removal and the potential for hardware failure.

The hook plate technique utilizes hardware in the form of a plate placed superiorly on the distal clavicle, with a hook engaging the inferior surface of the acromion to stabilize the joint. However, this technique is associated with high complications in some patients [6]. The button technique is currently the most widely used method, utilizing a double button combined with a MINAR or TightRope fixation system inserted via holes between the coracoid process and the distal clavicle. This method is also safe and has acceptable results [7,8]. This technique is classified as minimally invasive, making it more advantageous for patients. However, some studies reported that this technique needs special instruments and implants with higher direct costs than others. Then, from the perspective of surgical techniques, arthroscopy with TightRope is a promising option for preventing prolonged pain due to its minimally invasive nature, safer process, and shorter recovery time for returning to activities [2]. Still, this method needs a higher cost. On the other hand, the classical “Weaver-Dunn” procedure has begun to fall out of favor. This technique utilizes the coracoacromial ligament, which is transferred from the acromion to the distal clavicle, to replace the function of the AC ligament to stabilize the joint. Nevertheless, this technique has resulted in a high rate of deformity recurrence and a weaker and less rigid function of the AC joint, leading to its decreased popularity [6].

The SMB technique is a surgical approach for treating AC injuries that aims to achieve anatomical reduction and joint stability. This procedure generally involves making a straight line or a saber-cut incision to find the clavicle and coracoid process. The coracoid and clavicle are drilled in alignment to allow the insertion of “a button” device system, connecting one miniplate of three holes as a button with a non- absorbable suture, which stabilizes the joint without rigid fixation. This system is simple, safe, and affordable. The device is tightened to restore standard spacing and allow joint movement, then securing the ligaments and fascia [7].

**Fig. 4:**
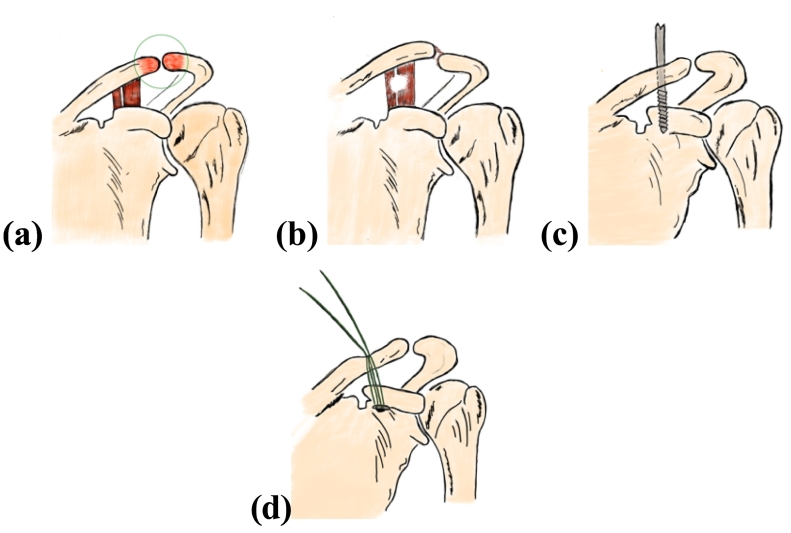
A 2-Dimension of AC joint injury area (a). A disruption of AC joint ligament (b). A hole was made using a bone drill (c). A single-button instrument was inserted (d).

This technique accurately restores the joint's anatomical alignment, thereby helping to decrease the risk of long-term complications such as post-traumatic arthritis or chronic instability. It employs a low-cost “button” and suture system that provides secure fixation, eliminating the need for more complex hardware, such as a titanium button, or using screws and hook plates, which may require removal after the healing process. Clinical outcomes have been positive, with many patients experiencing prompt recovery and regaining their previous pre-injury activity level [8]. However, according to Loriaut et al., a button method has some complications, such as infection, clavicle erosion, and secondary displacement, but it is still treatable (Fig. 4) [9].

Using button fixation, Boileau et al. reported 3,0 % recurrence seen 5 months after surgery [10]. Based on our patient, during this 6-month observation, no significant complaints were found after surgery. The patient was still advised to control at the surgery clinic until now to evaluate the healing progress.

Radiography is a standard modality for evaluating AC joint abnormalities, typically used at the beginning to diagnose and evaluate the treatment outcome. Our study showed a significant improvement in the X-ray appearance of the affected AC joint. There was a decline in the superior displacement of the clavicle in association with the superior border of the acromion after surgery, showing a promising result with the SMB technique.

Post-operative assessment of AC joint injury frequently utilizes the Visual Analog Scale (VAS) for pain evaluation [11]. A study by Saade and colleagues in 2023 found that the average VAS score in post-operative patients was lower than that of those treated conservatively [11,12]. Besides VAS, the Oxford Shoulder Score (OSS) is used for patients with AC joint injury following surgical treatment. It comprises 12 items that evaluate both shoulder pain and functionality post-operation and has been proven as a valid and reliable questionnaire [13]. Both VAS and OSS scores show improvement after treatment in this case.

## 4 Conclusion

In the case of AC joint injury, Rockwood classification grade III, a minimally invasive approach called the simple Modified Button Technique shows favorable results. However, long-term follow-up is still needed to prove this procedure.

## CRediT authorship contribution statement

All authors contributed to the creation of this case report.

## Consent

The patient provided written informed consent to publish this case report and its images. A copy of the consent form is available for review by the Editor- in-Chief upon request.

## Ethical approval

My institution did not require ethical approval for this case report.

## Guarantor

Romy Deviandri

## Sourcing of funds

This case report received no specific funding from public, commercial, or non- profit organizations.

## Declaration of competing interest

The authors declare that they have no conflicts of interest.
